# Deciphering the crosstalk of immune dysregulation between COVID-19 and idiopathic inflammatory myopathy

**DOI:** 10.3389/fimmu.2023.1197493

**Published:** 2023-08-10

**Authors:** Zhao Zhang, Weidong Tao, Debin Cheng, Marong Qin, Jun Fu, Dong Liu

**Affiliations:** ^1^ Department of Orthopaedics, Xi-Jing Hospital, The Fourth Military Medical University, Xi’an, China; ^2^ School of Chemistry, Cardiff University, Cardiff, United Kingdom

**Keywords:** COVID-19, idiopathic inflammatory myopathies, molecular mechanisms, immune dysregulation, biomarker

## Abstract

**Background:**

The coronavirus disease (COVID-19) pandemic is a serious threat to public health worldwide. Growing evidence reveals that there are certain links between COVID-19 and autoimmune diseases; in particular, COVID-19 and idiopathic inflammatory myopathies (IIM) have been observed to be clinically comorbid. Hence, this study aimed to elucidate the molecular mechanisms of COVID-19 and IIM from a genomic perspective.

**Methods:**

We obtained transcriptome data of patients with COVID-19 and IIM separately from the GEO database and identified common differentially expressed genes (DEGs) by intersection. We then performed functional enrichment, PPI, machine learning, gene expression regulatory network, and immune infiltration analyses of co-expressed genes.

**Results:**

A total of 91 common genes were identified between COVID-19 and IIM. Functional enrichment analysis revealed that these genes were mainly involved in immune dysregulation, response to external stimuli, and MAPK signaling pathways. The MCODE algorithm recognized two densely linked clusters in the common genes, which were related to inflammatory factors and interferon signaling. Subsequently, three key genes (CDKN1A, IFI27, and STAB1) were screened using machine learning to predict the occurrence of COVID-19 related IIM. These key genes exhibited excellent diagnostic performance in both training and validation cohorts. Moreover, we created TF-gene and miRNA-gene networks to reveal the regulation of key genes. Finally, we estimated the relationship between key genes and immune cell infiltration, of which IFI27 was positively associated with M1 macrophages.

**Conclusion:**

Our work revealed common molecular mechanisms, core genes, potential targets, and therapeutic approaches for COVID-19 and IIM from a genomic perspective. This provides new ideas for the diagnosis and treatment of COVID-19 related IIM in the future.

## Introduction

COVID-19 is a severe contagious illness caused by severe acute respiratory syndrome coronavirus 2 (SARS-CoV-2), which primarily spreads through respiratory droplets (1, 2). Since its emergence in Wuhan at the end of 2019, it has become an epidemic that affects public health worldwide, infecting billions of people (3). COVID-19 is a multisystem disease involving the respiratory, digestive, and musculoskeletal systems (**Table 1**) (4). The most common symptoms are fever, cough, myalgia, dyspnea, and fatigue (5). Increased research has shown that patients with COVID-19 have several immunological abnormalities that resemble those of autoimmune diseases (6, 7). SARS-CoV-2 infection can cause chronic inflammatory and immune responses that not only directly mediate tissue damage but may also induce serious sequelae of autoimmune disease in susceptible populations (8–10). With the increasing number of people recovering from SARS-CoV-2 infection, the connection between COVID-19 and autoimmune diseases is gaining significance.

**Table 1:** The summary of symptoms of COVID-19 affecting the different systems.

System	Symptom
Respiratory System	cough, dyspnea, fever, chest distress, thoracodynia
Cardiovascular System	palpitation, thoracodynia, myocardial injury, thrombus, pulmonary embolism
Digestive System	diarrhea, nausea, vomit, celialgia, abnormal liver function, liver injury
Nervous System	headache, fatigue, dysolfaction, dysgeusia, apoplexy, meningitis, encephalitis
Endocrine System	thyroid dysfunction, insulin resistance, hyperglycemia, hypophysial dysfunction
Musculoskeletal System	myalgia, fatigue, joint pain, muscle weakness, muscle injury

Idiopathic inflammatory myopathies (IIM), also known as myositis, represent a group of autoimmune muscle diseases with striking heterogeneity, characterized by myositis, progressive muscle weakness, and inflammatory cell infiltration of the muscle, along with other visceral organ damage (11). Based on clinical serology and pathomorphological features, IIM can be categorized into several disease subtypes, including nonspecific myositis, dermatomyositis, polymyositis, inclusion body myositis and necrotizing myopathy (12). Although there has been dramatic progress in the classification and treatment of IIM, the specific pathogenesis of IIM has not yet been fully elucidated due to the multifactorial nature of the disease. Like other types of autoimmunity, IIM is thought to be a consequence of the interaction between genetic and environmental risk factors in the absence of protective factors (13). Dysregulation of the immune system, especially in the genetic regions of human leukocyte antigens (HLA), has been recognized as an essential genetic risk factor for IIM (14). In addition, adaptive and innate immune mechanisms participate to varying degrees in different subtypes of IIM.

Notably, viral infection and vaccination have been established as critical triggers of autoimmunity in patients with IIM (15–18). Patients with COVID-19 frequently exhibit immune dysregulation, which can contribute to multiple autoimmune diseases, including IIM (19). Kharouf et al. observed that the incidence of IIM increased significantly during the COVID-19 pandemic and that these patients exhibited unique characteristics and more severe symptoms (20). The presentation of COVID-19 induced myositis may vary from significant muscle weakness to typical dermatomyositis or simply back pain with muscle disease. The pathophysiological process of the hyperinflammatory response triggered by COVID-19, leading to extensive endothelial dysfunction, vasculopathy, and thrombosis is highly similar to anti-MDA5 myositis (21). A few case reviews have also reported the risk of anti-MDA5 myositis shortly after COVID-19 vaccination (22, 23). Moreover, patients with autoimmune diseases are more susceptible to SARS-CoV-2 infections. IIM patients are more readily hospitalized for COVID-19 all-cause than healthy individuals, the course of COVID-19 is more severe, and disease activity and drug exposure are strongly linked to its severity of COVID-19 (24, 25). Additionally, following recovery from acute SARS-CoV-2 infection, a large proportion of patients develop a range of persistent symptoms and complications, also known as Long COVID (26). Long COVID accumulates in all systems and organs of the body, with common symptoms including fatigue, shortness of breath, dyspnea, muscle pain, and joint pain. Certain symptoms are typical of IIM, causing serious physical and mental health impacts (27). Growing evidence suggests that Long COVID seems to be an autoimmune disease in which the infected patient produces an “autoantibody” that attacks their own tissues, resulting in a chronic, persistent inflammatory response in recovering patients (28). Although the link between COVID-19 and autoimmune diseases has begun to attract the attention of researchers, few studies have shed light on the common molecular mechanisms of COVID-19 and IIM.

Here, we aimed to decipher the crosstalk between COVID-19 and IIM from a genetic perspective using bioinformatics analysis. Moreover, we applied machine learning to identify key biomarkers for the occurrence of COVID-19-related IIM and evaluated the effect of these markers on immune infiltration of IIM. These studies provide a deeper understanding of the comorbidity mechanisms of COVID-19 and IIM.

## Materials and methods

### Data collection and processing

The COVID-19 and IIM datasets (GSE171110, GSE128470, and GSE39454) were retrieved from the GEO database. The GSE171110 dataset consisted of whole-blood gene expression profiles of 44 COVID-19 patients and 10 healthy controls (HC). The GSE128470 dataset included gene expression profiles of muscle tissues from 65 patients with IIM and 12 HCs. The GSE39454 dataset included gene expression profiles of muscle tissues from 31 patients with IIM and five HCs. Subsequently, the R package “limma” was used to analyze differentially expressed genes (DEGs). In the GSE171110 dataset, |log_2_FoldChange| ≥1 and |adj.P.Val.| <0.05 were set as the threshold. The GSE128470 dataset was used as the test set, with |log2FoldChange| ≥0.58496 and |adj.P.Val.| <0.05 set as the threshold value. The GSE39454 dataset was used as a validation cohort to verify key diagnostic genes. Subtype classifications of the IIM cohort are summarized in **Table 2**.

**Table 2:** Summary of subtype classification of the IIM cohort.

Subtype	GSE128470	GSE39454
dermatomyositis	12	8
polymyositis	7	8
inclusion body myositis	26	10
necrotizing myopathy	6	5
nonspecific myositis	14	0

### Functional enrichment analysis

To determine the biological functions and pathways involved in common genes, we performed and visualized Wikipathway, KEGG pathway, GO terms, and hallmark gene sets for common genes using the R package “clusterProfiler.”

### PPI analysis

A PPI network of common genes was established in STRING (version 11.0), with confidence scores >0.15 set as the threshold, and visualized using Cytoscape (29, 30). In addition, the Molecular Complex Detection (MCODE) algorithm was used to recognize densely linked network components in Metascape (31).

### Machine learning for the identification of key genes

To identify key biomarkers for the occurrence of COVID-19 related IIM, we used two machine learning methods for screening (LASSO and Random forest) (32–35). LASSO was performed by R package “glmnet,” and the minimum lamba value was set as the threshold. Random forest algorithm was executed with the R package “randomForest” with the relative importance score >0.3 set as a threshold. Finally, the intersection of the outputs of the two algorithms was considered as the key gene.

### Validation of key genes and evaluation of diagnostic performance

We further validated and evaluated the expression levels of these key genes in the GSE39454 cohort. Subsequently, ROC curves were used to evaluate the diagnostic performance of these key genes by the R package “pROC.”

### Constructing regulatory networks of transcription factors and miRNAs of key genes

Transcription factors and miRNAs play essential roles in the regulation of gene expression (36). Subsequently, we established TF-gene and miRNA-gene networks based on the NetworkAnalyst database (https://www.networkanalyst.ca/) (37).

### Immune infiltration analysis

The CIBERSORT algorithm was used to determine the infiltration abundance of 22 immune cells in each sample of the IIM cohort. Spearman’s correlation analysis was used to assess the relationship between key genes and immune cells.

### Scanning for candidate agents

The DSigDB tool was utilized to screen for candidate agents interacting with key genes (38). The top 11 agents were identified using p-values.

### Statistics

R 4.0.5 software and SPSS 21.0 software were used for statistical analysis. Wilcoxon was employed to determine the differences between groups, and p-value <0.05 was set as the threshold (*p <0.05, **p <0.01, ***p <0.001).

## Result

### Identification of DEGs in COVID-19 and IIM

**Figure 1:**
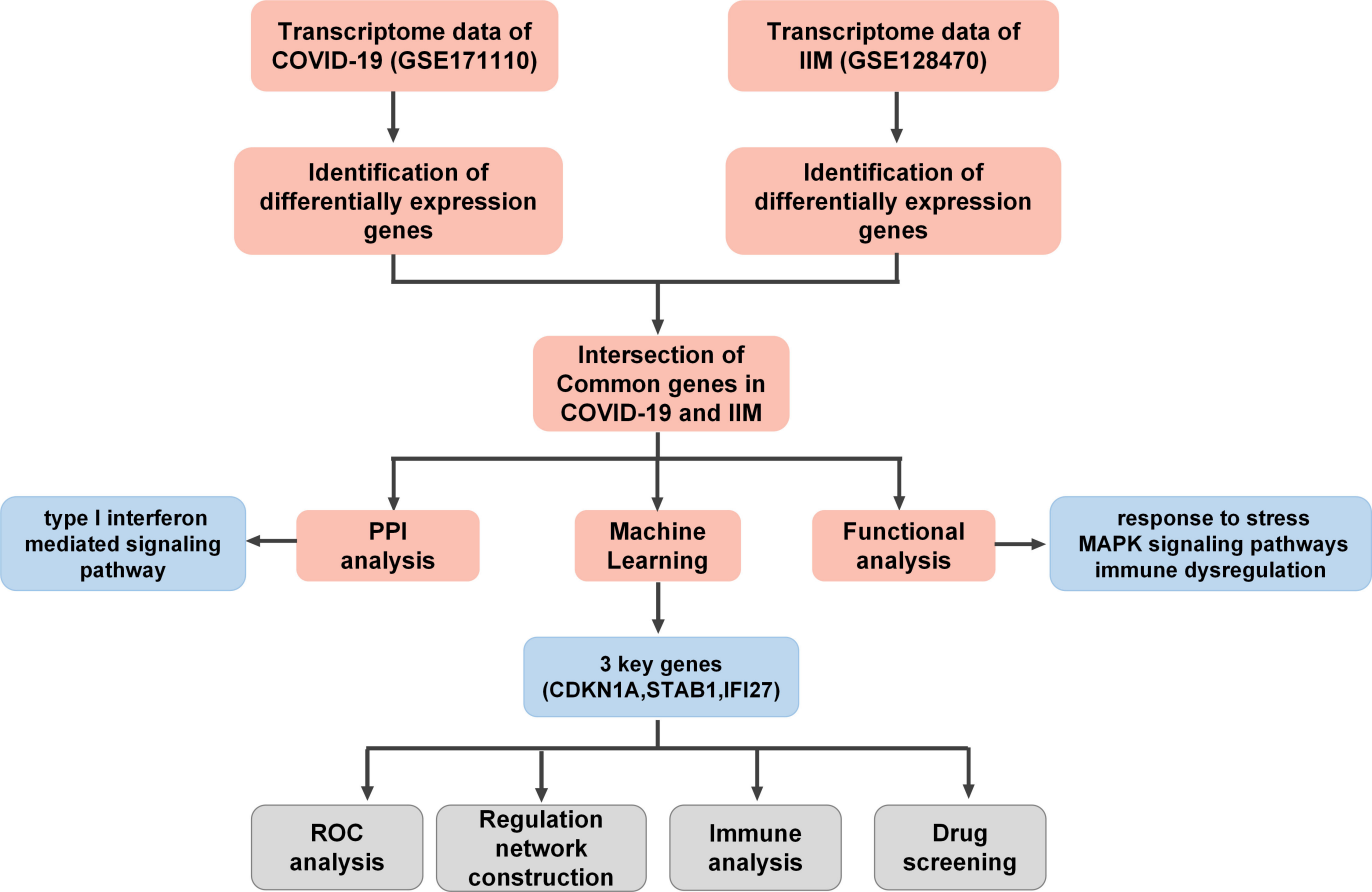
Flow chart of this study.

**Figure 2:**
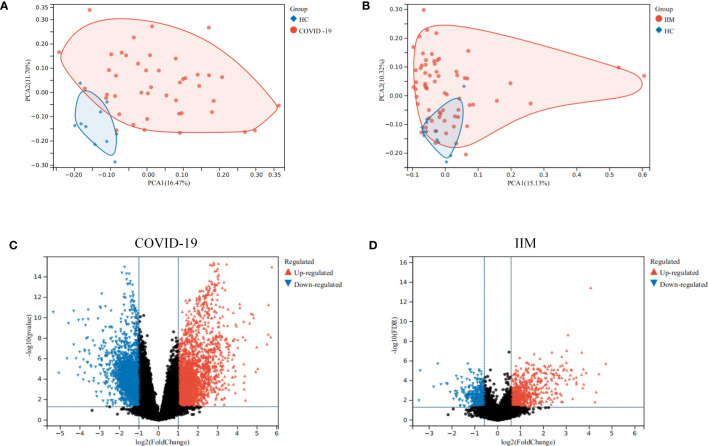
Analysis of DEGs in patients with COVID-19 and IIM. **(A)** PCA plot for the COVID-19 dataset; **(B)** PCA plot for the IIM dataset; **(C)** volcano diagram of DEGs in the COVID-19 dataset; **(D)** volcano diagram of DEGs in the IIM dataset.

A flowchart of the study is presented in **Figure 1**. Using principal component analysis, we found that the disease and HC groups could be separated distinctly in the COVID-19 and IIM cohorts, respectively (**Figures 2A, B**). In the COVID-19 dataset, 3,803 DEGs were identified, of which 2,020 were upregulated and 1,783 were downregulated (**Figure 2C**). In the IIM dataset, 1,040 DEGs were identified, including 650 upregulated and 390 downregulated genes (**Figure 2D**).

### Confirmation of common genes

**Figure 3:**
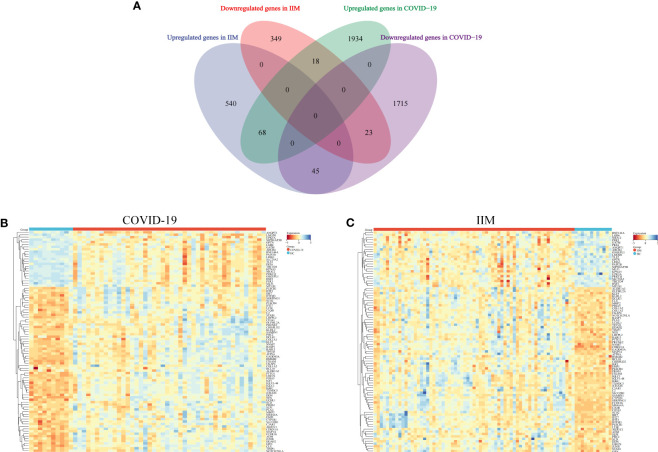
Identification of common molecules in COVID-19 and IIM patients. **(A)** Intersection of the COVID-19 and IIM DEGs; **(B)** heat map of common gene expression in the COVID-19 dataset; **(C)** heat map of common gene expression in the IIM dataset.

Subsequently, Wayne analysis was performed to intersect DEGs in the two datasets (**Figure 3A**). The results revealed 68 common upregulated expression genes and 23 common downregulated expression genes between the two diseases (**Table S1**). Heatmaps showing the expression levels of these genes in the COVID-19 and IIM cohorts, respectively (**Figures 3B, C**).

### Functional enrichment analysis

**Figure 4:**
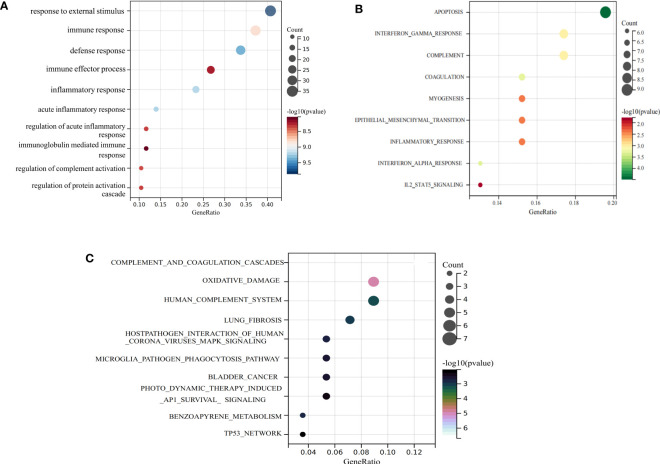
Functional enrichment analysis of common genes. **(A)** GO term enrichment; **(B)** hallmark gene set enrichment; **(C)** Wikipathway enrichment.

We performed an enrichment analysis of these common genes to reveal the comorbidity mechanism between COVID-19 and IIM. GO terms found that these common genes were mostly involved in response to external stimuli, immune response, defense response, and other functions; hallmark gene sets found that these common genes were predominantly involved in apoptosis, interferon gamma response, complement and so on; Wikipathways identified these common genes as involved in complement, and coagulation cascades, oxidative damage, host–pathogen interaction of human corona viruses MAPK signaling, etc. (**Figure 4**).

### PPI network

**Figure 5:**
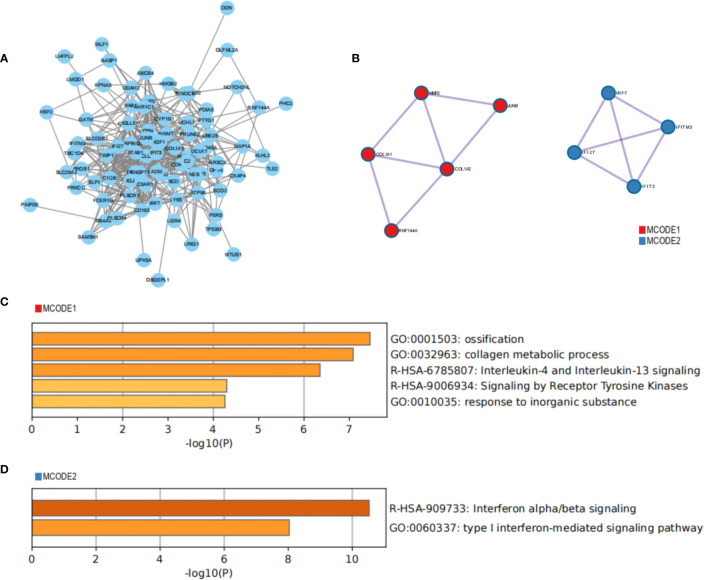
Construction of PPI network to identify core clusters. **(A)** PPI network of the common genes; **(B)** MCODE algorithm recognizes core clusters; **(C, D)** functional enrichment analysis of core clusters.

A PPI network of common genes was constructed using Cytoscape, which contained 89 nodes and 460 edges (**Figure 5A**). Moreover, the MCODE algorithm recognized two densely linked clusters (**Figure 5B**). Cluster 1 was mostly involved in ossification, collagen metabolic process and lnterleukin-4 and Interleukin-13 signaling; Cluster 2 was primary involvement in defense response to virus and type I interferon-mediated signaling pathway (**Figures 5C, D**).

### Comorbidity mechanisms between the different IIM subtypes and COVID-19

Given that the interferon pathway is known to be significantly upregulated in dermatomyositis, to reduce the bias of our results, we divided the IIM patients into subgroups of patients with and without dermatomyositis for further analysis. A total of 68 common upregulated genes and 11 common downregulated genes were identified between dermatomyositis and COVID-19 (**Table S2**), in which two densely linked clusters were identified (**Figures S1A, B**). Cluster 1 was correlated with defense response to virus, type I interferon-mediated signaling pathway, negative regulation of viral genome replication and organelle inner membrane; Cluster 2 was related to protein processing in endoplasmic reticulum (**Figure S1C**). In addition, 64 common upregulated genes and 29 common downregulated genes were identified between other IIMs and COVID-19 (**Table S3**), in which two densely linked clusters were also identified (**Figures S1D, E**). Cluster 1 was mostly involved in proteoglycans in cancer, ossification, regulation of vascular associated smooth muscle cell proliferation, and regulation of lymphocyte activation; Cluster 2 was related to defense response to virus and type I interferon-mediated signaling pathway (**Figure S1F**). These findings reduce the bias of our analysis and further increase the robustness of the results.

### Machine learning identified key genes in COVID-19 related IIM

**Figure 6:**
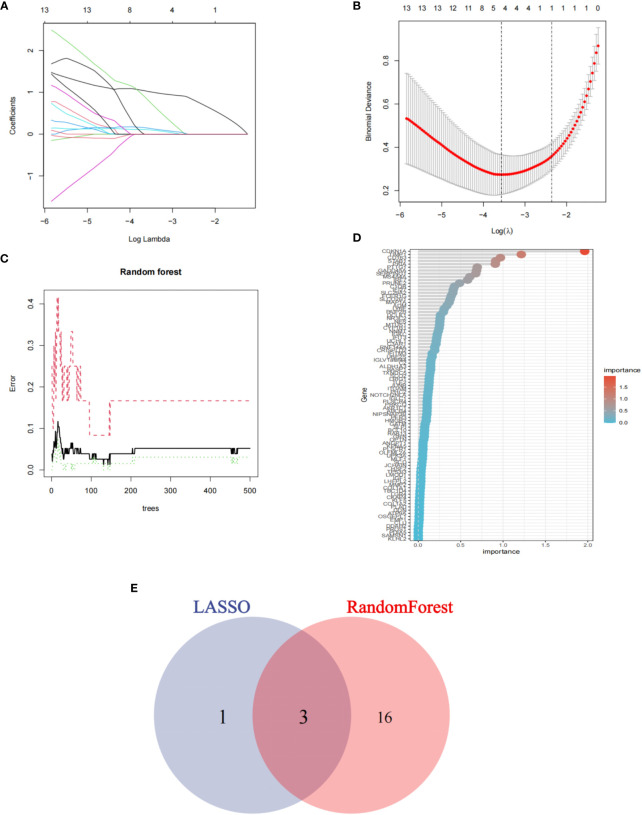
Machine learning was used to identify key genes. **(A, B)**. LASSO algorithm; **(C, D)** Random forest algorithm; **(E)** Intersection between the two algorithms.

To identify the key genes involved in the occurrence of COVID-19 related IIM, we screened for common genes using two machine learning methods. The LASSO algorithm confirmed four common genes and the random forest algorithm confirmed 19 common genes (**Figures 6A–D**). Finally, we intersected the two algorithms and obtained a total of four genes, which were considered key genes for COVID-19 related IIM (**Figure 6E**).

### Validation of key genes and diagnostic performance evaluation

**Figure 7:**
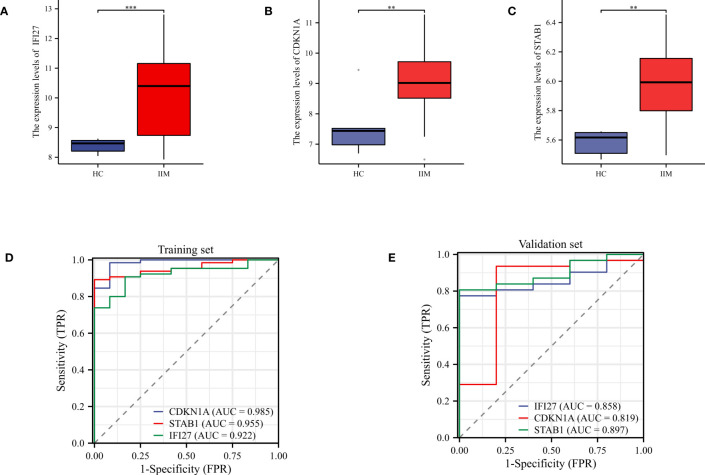
Validation of key genes and evaluation of diagnostic performance evaluation. **(A–C)** Expression levels of key genes in the validation set; **(D, E)** ROC curves were used to evaluate the diagnostic performance of the key genes in the training and validation sets. **p < 0.01, and ***p < 0.001.

Subsequently, we verified the expression of key genes in the validation set. The expression levels of these key genes were elevated in IIM compared to HC, which was consistent with the results of the test set (**Figures 7A–C**). ROC curves revealed that the AUC values of these genes were higher than 0.8 in both the training and validation sets, indicating that these genes exhibited excellent diagnostic performance (**Figures 7D, E**).

### Construction of TF-key gene and miRNA-key gene networks

**Figure 8:**
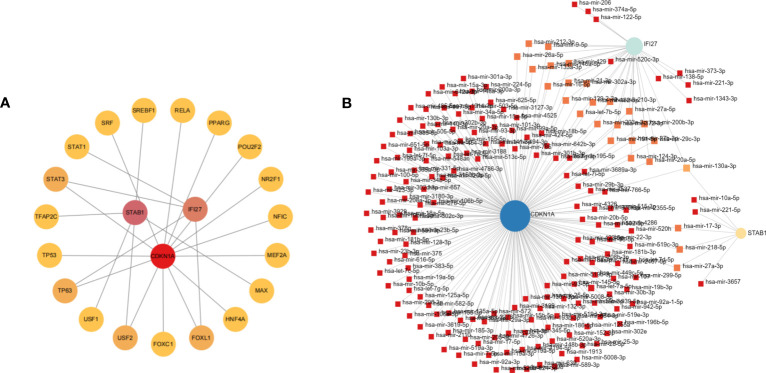
Building a regulatory network for key gene expression. **(A)** TF-genes networks; **(B)** miRNA-gene networks.

To reveal the regulatory mechanisms of key genes, we predicted the upstream and downstream TFs and miRNAs of key genes. It was revealed that 14 TFs can regulate CDKN1A, five TFs can regulate STAB1, four TFs can regulate IFI27 (**Figure 8A**). As for miRNAs, we found that 187 miRNAs could interact with CDKN1A, 31 miRNAs could interact with IFI27, and seven miRNAs could interact with STAB1 (**Figure 8B**). TFs can regulate the expression of key genes by binding to specific DNA sequences to control the transcription of target genes. MiRNAs are important post-transcriptional regulators that regulate key gene expression by binding to specific messenger RNA (mRNA) molecules, which can lead to mRNA degradation or inhibit protein translation. Our results provide new insights into the upstream and downstream regulatory mechanisms of key gene expression levels.

### Immune infiltration analysis

**Figure 9:**
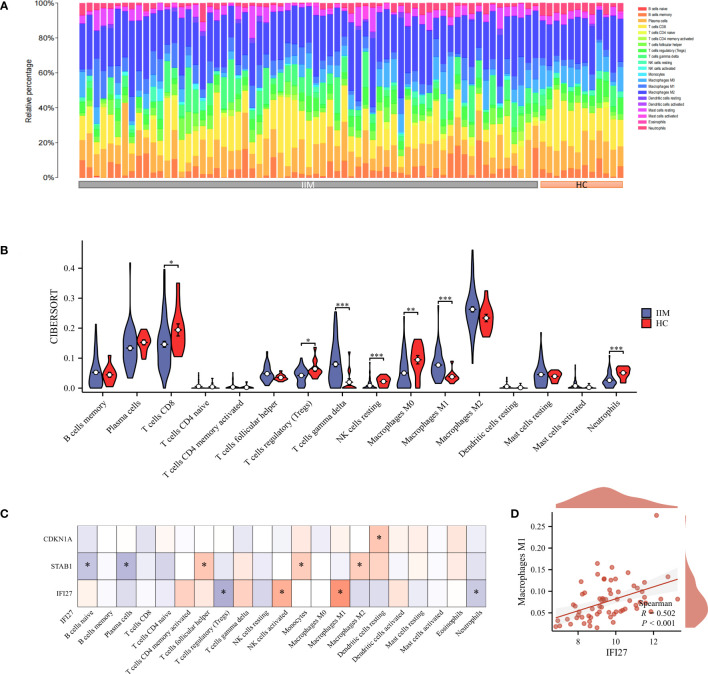
Immuno-infiltration analysis. **(A, B)** Abundance of 22 immune-infiltrating cells in IIM; **(C)** Correlation heat map of key genes and immune-infiltrating cells; **(D)** Correlation scatter plot of key genes with M1 macrophages. *p < 0.05, **p < 0.01, and ***p < 0.001.

Considering that immune cell infiltration is an essential feature in the pathogenesis of IIM, we estimated the infiltration abundance of 22 immune cell types in the immune microenvironment of IIM using the CIBERSORT algorithm (**Figure 9A**). The results revealed that T cell gamma delta and M1 macrophage showed markedly increased infiltration abundance in IIM, whereas T cell CD8, Tregs, NK cell resting, M0 macrophage, and neutrophils exhibited opposite results (**Figure 9B**). Correlation analysis showed that the key genes were related to different infiltrating immune cells (**Figure 9C**). In particular, IFI27 was positively linked to M1 macrophage, which is considered an active mediator of virus infection-associated myositis (**Figure 9D**).

### Screening of candidate agents

To identify potential agents for the treatment of COVID-19 related IIM, we predicted the agents that interact with these key genes using the DSigDB database. We found that 11 agents interacted with CDKN1A, five agents interacted with IFI27, and 1 agent interacted with STAB1 (**Table 3**).

**Table 3:** Agent candidates interacted with key genes.

Agent	P-value	Combined Score	Genes
tamoxifen	2.49E−04	598.0306794	CDKN1A;IFI27;STAB1
cinnamaldehyde	4.02E−04	951.5310143	CDKN1A;IFI27
Zinc sulfate	0.001207993	464.6813047	CDKN1A;IFI27
0175029-0000	0.001976804	333.9392188	CDKN1A;IFI27
3’-Azido-3’-deoxythymidine	0.002041055	326.765372	CDKN1A;IFI27
L-mimosine	0.002198311	4077.18792	CDKN1A
MCDF	0.002198311	4077.18792	CDKN1A
Dimethyl-IQX	0.002397979	3653.699525	CDKN1A
Thymoquinone	0.002397979	3653.699525	CDKN1A
64551-89-9	0.002397979	3653.699525	CDKN1A
gamma-Tocopherol	0.002397979	3653.699525	CDKN1A

## Discussion

In the present study, we identified 86 common genes between COVID-19 and IIM using differential expression analysis, of which 56 were upregulated and 16 were downregulated. Functional enrichment analysis of common genes showed that these genes were primarily linked to response to stress, MAPK signaling pathways and immune dysregulation-related pathways such as complement and coagulation cascades, interferon response, and others. MAPK pathways have been recognized as an efficient transmitter of signals from the cell surface to the nucleus. MAPK can respond to different extracellular stimuli and thus generate contingency responses to trigger cascade signals to regulate cell proliferation, differentiation, and apoptosis (39, 40). Further analysis revealed that two closely linked clusters of common genes were related to inflammatory factors and interferon signaling. During SARS-CoV2 infection, the body produces large amounts of inflammatory mediators and chemokines that recruit immune cell infiltration to mediate inflammatory injury (41). An over-activated immune response of the organism induces a cytokine storm, which is positively related to the severity of COVID-19 (42). Immune dysregulation, especially interferon response, is considered as an essential sign of the progression and worsening of COVID-19 and IIM (43, 44). Interferons are potent cytokines and components of the first line of defense against viral infections (45). According to their distinct molecular characteristics and intracellular recognition receptors, interferons are classified as types I, II, and III, which induce hundreds of interferon-stimulated effector genes (ISGs) with dual roles in antiviral and immunomodulatory functions (46). Both the early stages of interferons deficiency and the late stages of interferons persistence can be indicators of severe COVID-19 (47, 48). Similarly, apart from genetic susceptibility and environmental factors, adaptive and innate immune mechanisms have recently been shown to be involved in the pathogenesis of IIM via coordinated interactions (49). Interferon signaling is highly represented in the muscle and skin of IIM patients, together with complement cascade activation, exerting various inflammatory effects that result in muscle fibrosis (50). In particular, interferon I signaling is increased in anti-MDA5+ dermatomyositis, which greatly correlates with disease activity and can be used to predict patient mortality (51, 52). Therefore, we hypothesized that after COVID-19 infection, myocytes respond to external stimuli to activate complement and interferon signaling via MAPK signaling, which releases large amounts of inflammatory factors. Ultimately, persistent inflammatory muscle damage can result in the development of IIM.

The extrapulmonary manifestation of COVID-19 is thought to occur through ACE-2 receptor-mediated viral attack (53). Similar to most other SARS-CoV-2 susceptible regions, the muscle tissue also highly expresses ACE-2 receptors (54). Viral stinger proteins can attach to the ACE-2 receptor, allowing the SARS-CoV-2 viral envelope to bind to the host cell membrane and transfer hereditary materials into the cell to strike it (55). This indicates that SARS-CoV-2 may be directly responsible for infecting muscle cells to activate the immune response (56). Altogether, the integration of our results may provide a new perspective for understanding the common pathogenesis of COVID-19 and IIM.

To shed further light on the key targets for the emergence of COVID-19-associated IIM, we used machine learning to screen four key genes for predicting the occurrence of COVID-19 related IIM. Interferon alpha-inducible protein 27 (IFI27) acts upstream of the negative regulation of RNA polymerase II transcription and regulates the export of proteins from the nucleus. It is involved in several cellular processes that mediate cytokine signaling in innate and adaptive immunity (57). An observational multi-cohort study found that IFI27 is highly expressed in the lower respiratory airways of COVID-19 patients and is associated with the presence of a high viral load (58). The upregulation of IFI27 expression in blood could be a predictor of respiratory failure in COVID-19 patients. The aberrant expression of IFI27 is also involved in the development of several autoimmune diseases (59). CDKN1A encodes a potent cyclin-dependent kinase inhibitor that functions in DNA damage repair and execution of apoptosis after caspase activation. CDKN1A is markedly upregulated in COVID-19 dependent muscle loss (60). Wang et al. (61) revealed that CDKN1A can be used for the early diagnosis of dermatomyositis as well as promoting the development of dermatomyositis by regulating immune cell infiltration. STAB1 is a type l transmembrane receptor, also known as a multifunctional scavenger receptor, that is highly expressed on macrophage endothelial cells (62). STAB1 mediates endocytose ligands and is critical for cell adhesion in chronic inflammation and tumor metastasis (63). Sotzny et al. (64) found that STAB1 could stratify patients with post-COVID syndrome and predict the development of myalgic encephalomyelitis/chronic fatigue syndrome. ROC curves showed that these genes exhibited high diagnostic value for the development of IIM and may be expected to be potential targets for intervention in COVID-19 related IIM.

Transcription factors and miRNAs are pivotal factors that regulate gene expression in organisms and are involved in transcriptional regulation and post-transcriptional regulation of genes, respectively (36). We constructed an interaction network to elucidate the regulatory network of TFs and miRNAs involving key genes, which revealed upstream and downstream expression regulation of key genes. Moreover, we characterized the immune microenvironment of IIM. Consistent with previous findings, there was a substantial infiltration of pro-inflammatory immune cells in the IIM group (follicular helper T cells, gamma delta T cells, and M1 macrophages) and a clear lack of suppressive inflammatory cells (Tregs) compared to the HC group (65–67). Correlation analysis revealed that key genes were linked to multiple immune-infiltrating cells, indicating that key genes may be involved in the progression of IIM by regulating immune cells. Notably, the expression level of IFI27 was positively correlated with M1 macrophage. Macrophages can be classified into classically activated M1 macrophages and selectively activated M2 macrophages (68). M1 macrophages are generally polarized by interferon-gamma, secrete large amounts of pro-inflammatory factors, and play an essential role in the early stages of inflammation. M2 macrophage express suppressive inflammatory factors that inhibit the inflammatory response and tissue repair (34, 35). Watson et al. (69) showed that M1 macrophage were essential mediators of virus-induced myopathy and that blocking SHP-1 activation in macrophages could prevent virus-induced myofiber degeneration. Finally, several potential agents which interact with these key genes were screened. These agents mainly exert their therapeutic effects by inhibiting the release of inflammatory mediators and modulating immune cell activity. Thus, we suggest that these agents target key genes in the treatment of COVID-19 related IIM. Collectively, our findings provide a comprehensive analysis to understand the pathogenesis of these key genes in COVID-19 related IIM.

This study had some limitations. First, this study was based on the bioinformatics analysis of public databases, and extensive experiments are necessary to validate the value of these molecules, pathways, and drugs. Second, this study was a retrospective analysis based on a public database, with a small amount of data and the absence of clinical information. Many patients, especially those infected with SARS-CoV-2 and IIM, will need to be acquired in the future to further validate the clinical applicability of our findings.

## Conclusion

In conclusion, our study revealed for the first time the critical role of MAPK signaling and inflammatory responses in the link between COVID-19 and IIM comorbidity development. Moreover, four key genes were screened using machine learning for early diagnosis and treatment of COVID-19 related IIM. These findings provide thorough insight into the pathogenesis of comorbid IIM in patients with COVID-19. As the COVID-19 pandemic continues, a great deal of work is needed in the future to focus on the crosstalk between COVID-19 and IIM.

## Data availability statement

The original contributions presented in the study are included in the article/**Supplementary Material**. Further inquiries can be directed to the corresponding authors.

## Author contributions

Conception: DL and JF. Interpretation or analysis of data: ZZ, WT, and DC. Preparation of the manuscript: ZZ, WT, and DC. Revision for important intellectual content: MQ. Supervision: DL and JF. All authors listed have made a substantial, direct, and intellectual contribution to the work and approved it for publication.
