# Supplementary material for: Deciphering the crosstalk of immune dysregulation between COVID-19 and idiopathic inflammatory myopathy
Source: Front Immunol. 2023 Aug 10;14:1197493. doi: 10.3389/fimmu.2023.1197493 (PMC10449257; doi:10.3389/fimmu.2023.1197493)
Supplement: Supplementary file 1 [file DataSheet_1.docx]

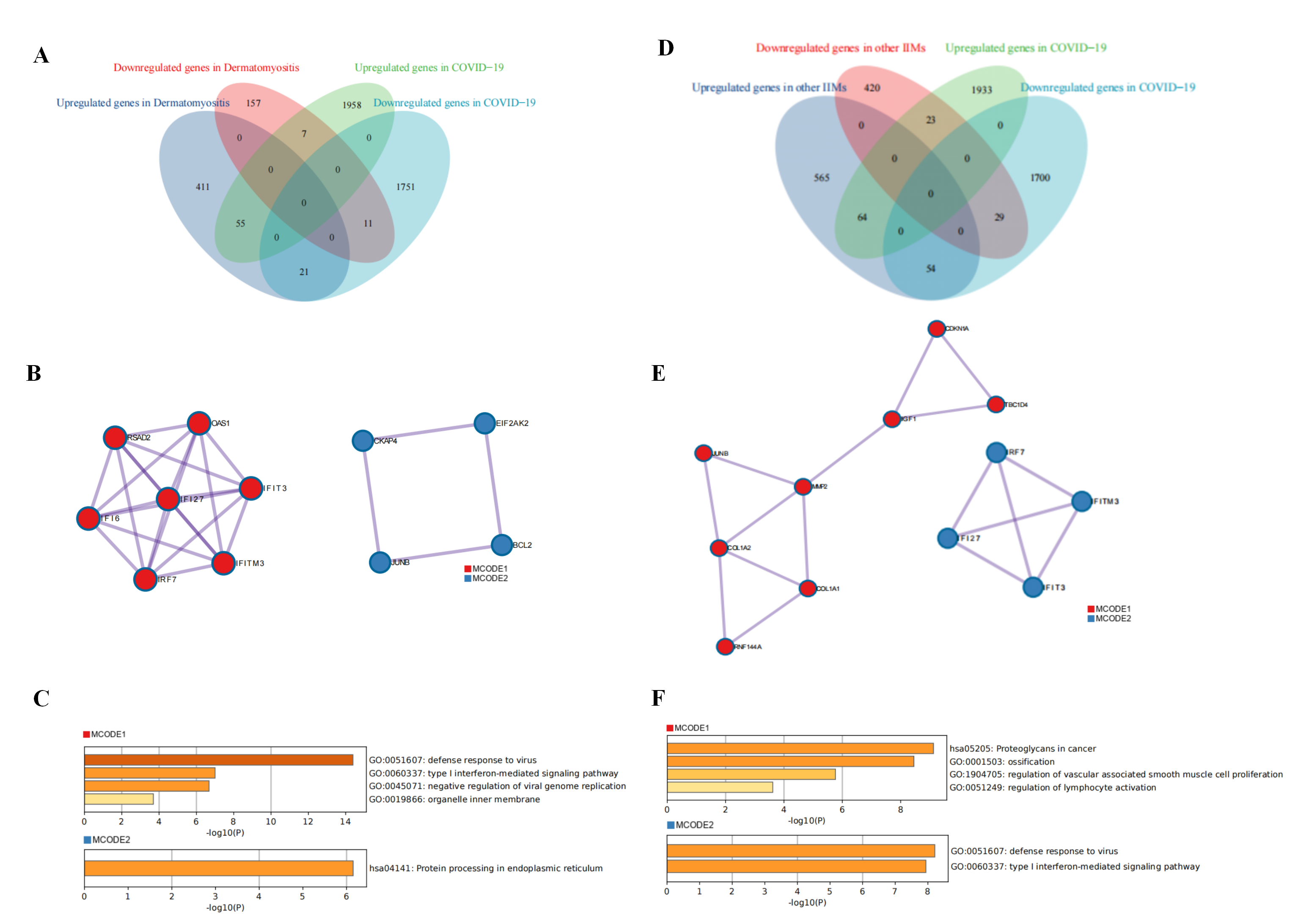


**Figure S1.** Co-morbidity mechanisms between the different IIM subtypes and COVID-19. (A). Common DEGs between dermatomyositis and COVID-19; (B). MCODE algorithm recognition core Cluster between dermatomyositis and COVID-19; (C). Functional enrichment analysis of core Cluster between dermatomyositis and COVID-19; (D). Common DEGs between other IIM and COVID-19; (E). MCODE algorithm recognition core Cluster between other IIM and COVID-19; (F). Functional enrichment analysis of core Cluster between other IIM and COVID-19;
